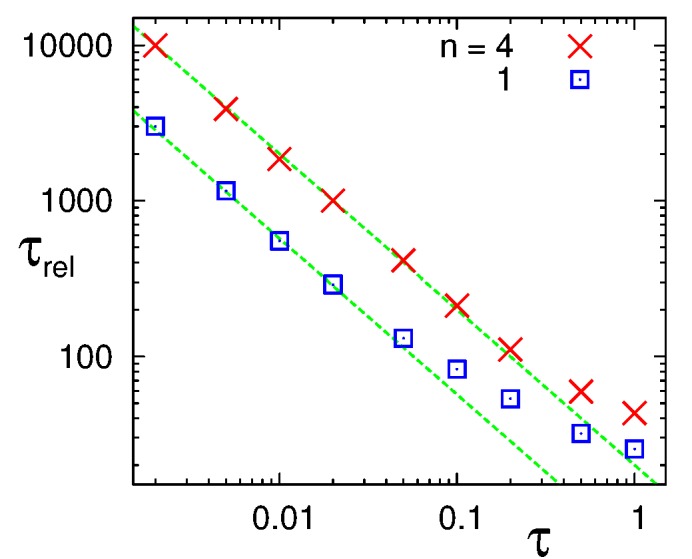# Correction: Transcription Fluctuation Effects on Biochemical Oscillations

**DOI:** 10.1371/annotation/23bca9d0-f934-400e-8bb9-f5ff07f9e625

**Published:** 2013-11-08

**Authors:** Ryota Nishino, Takahiro Sakaue, Hiizu Nakanishi

An error was introduced in the preparation of this article for publication. Figures 2-6 are not displayed correctly. Please find the correct versions of each figure below:

Figure 2: 

**Figure:**
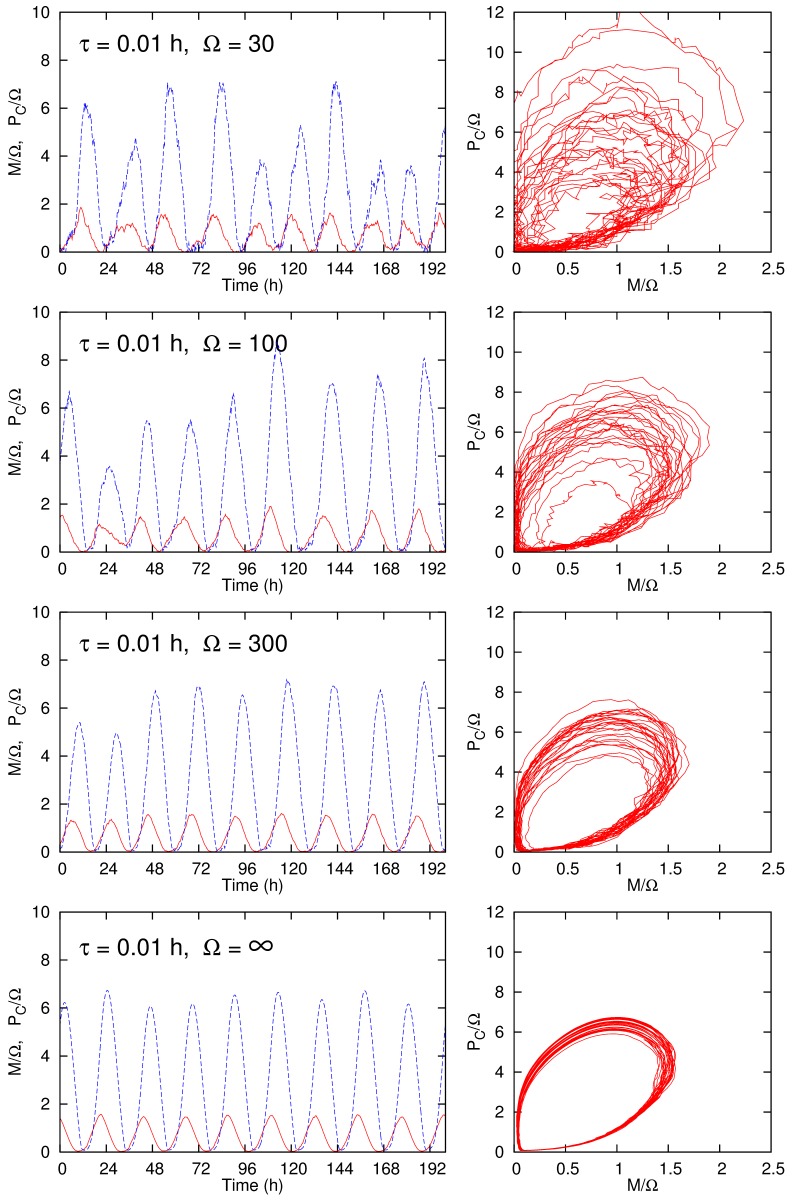


Figure 3: 

**Figure:**
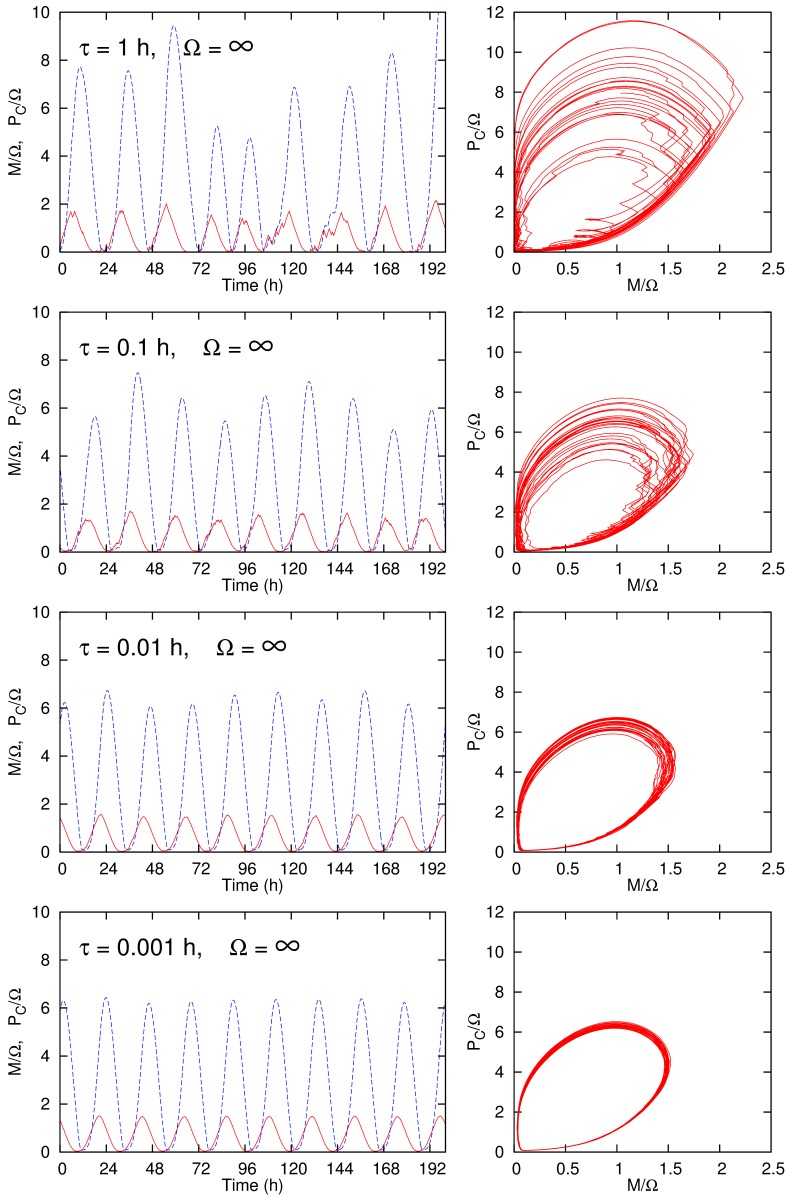


Figure 4: 

**Figure:**
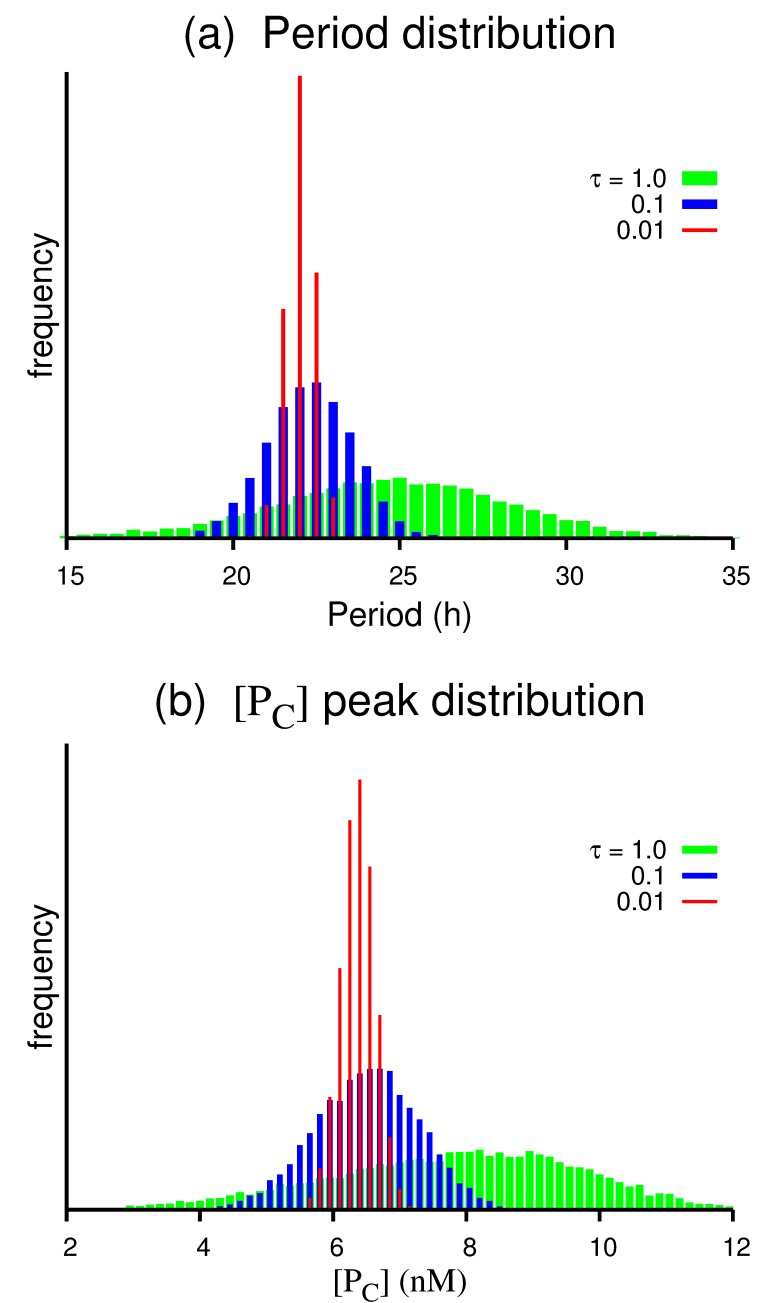


Figure 5: 

**Figure:**
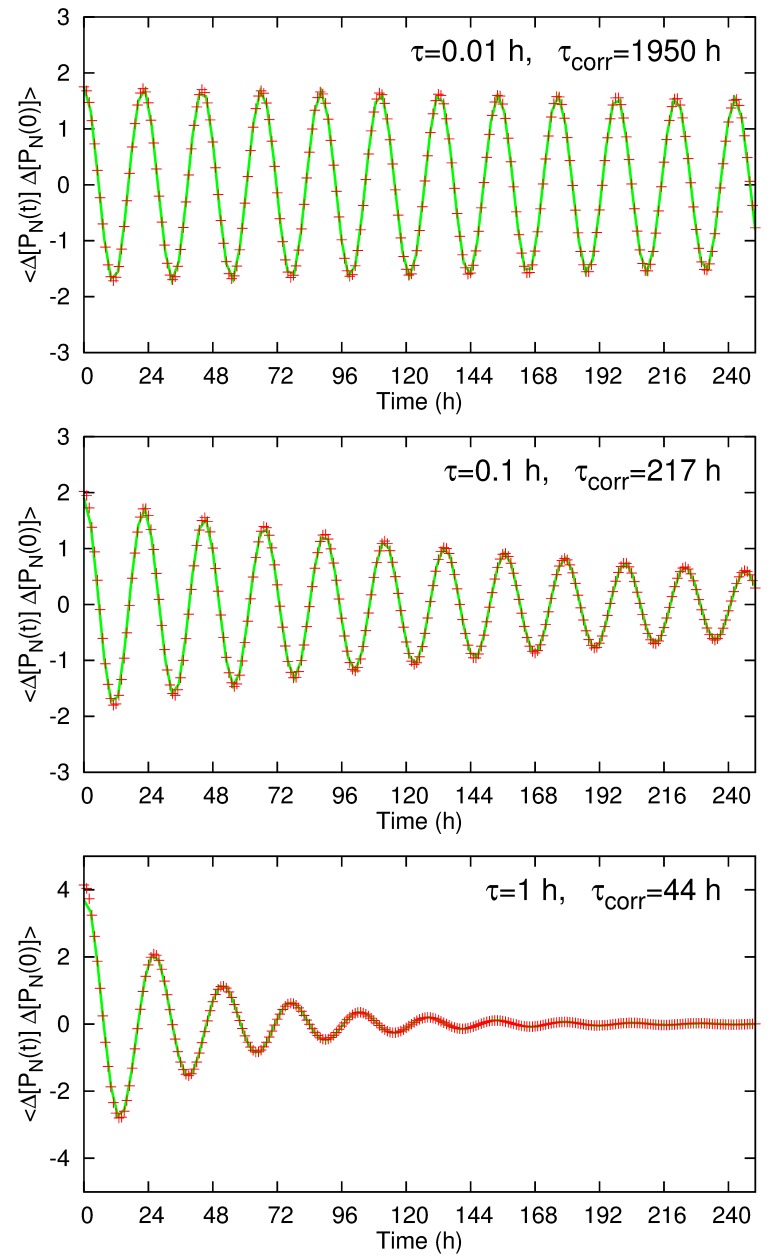


Figure 6: 

**Figure:**