# Correction: Transcription Fluctuation Effects on Biochemical Oscillations

**DOI:** 10.1371/journal.pone.0095119

**Published:** 2014-04-09

**Authors:** The PLOS ONE Staff

## Notice of Republication

This article was republished on March 20, 2014, to replace Figures 2-6 and Table 1, which were originally published incorrectly. This republication also resolves the initial correction published on November 8, 2013. The publisher apologizes for these errors. Please download this article again to view the correct version. The originally published, uncorrected article and the republished, corrected article are provided here for reference.

## Supporting Information

File S1 Originally published, uncorrected article.
(PDF)

File S2 Republished corrected article.
(PDF)
