# Supplementary material for: Transcription Fluctuation Effects on Biochemical Oscillations
Source: PLoS One. 2013 Apr 12;8(4):e60938. doi: 10.1371/journal.pone.0060938 (PMC3625213; doi:10.1371/journal.pone.0060938)
Supplement: Supporting Information S1 — Appendix. (PDF) [file pone.0060938.s001.pdf]

Transcription fluctuation effects on biochemical oscillations

Ryota Nishino[1], Takahiro Sakaue[1], Hiizu Nakanishi[1,*]
1 Department of Physics, Kyushu University, Fukuoka, Japan
∗ E-mail: Corresponding nakanisi@phys.kyushu-u.ac.jp

Appendix: Phase diffusion and correlation time

In the appendix, we show that the correlation time $\tau_{\rm corr}$ in the correlation function is proportional to $1/\tau$ when the period distribution has the width proportional to $\sqrt{\tau}$.

Suppose the correlation function $C(t)$ is written as

$$C(t) = A \int_{-\infty}^{\infty} \cos\big(\omega_0 t + \theta\big) P(\theta, t) d\theta \tag{A.1}$$

in terms of the average over the phase difference θ by the distribution function $P(\theta, t)$ at time t. Here, ω_0 is the average angular velocity given by $\omega_0 = 2\pi/T_0$ in terms of the average period T_0. Now, we assume that the phase distribution can be approximated by the Gaussian distribution with the standard deviation $\sigma_\theta(t)$,

$$P(\theta, t) \approx \frac{1}{\sqrt{2\pi\sigma_\theta^2(t)}} \exp\left[-\frac{\theta^2}{2\sigma_\theta^2(t)}\right], \tag{A.2}$$

then, Eq.(A.1) may be estimated as

$$C(t) \approx A \cos(\omega_0 t) \exp\left[-\frac{1}{2}\sigma_\theta^2(t)\right]. \tag{A.3}$$

Now, we estimate the phase distribution $P(\theta, t)$ as follows. The phase θ at the time $t = nT_0$ may be expressed as the sum of n phases accumulated by the time:

$$\theta = \sum_{i=1}^{n} 2\pi \left(\frac{1}{T_i} - \frac{1}{T_0}\right) T_0 \approx -\sum_{i=1}^{n} 2\pi \frac{\Delta T_i}{T_0}, \tag{A.4}$$

where T_i is the i'th period (i.e. peak-to-peak interval) with $T_i = T_0 + \Delta T_i$, and we have assumed $\Delta T_i \ll T_0$ in the last approximation.

If there is no correlation among ΔT_i, then $\sigma_\theta(t)$ is given by

$$\sigma_\theta^2(t) \approx \left(2\pi \frac{\sigma_T}{T_0}\right)^2 \frac{t}{T_0}, \tag{A.5}$$

where σ_T is the standard deviation of the period T. We have replaced n by t/T_0.

With Eq.(A.3), this gives the form of Eq.(19) with $\theta_0 = 0$ and

$$\tau_{\rm corr} = \left(\frac{1}{2\pi} \frac{T_0}{\sigma_T}\right)^2 2T_0, \tag{A.6}$$

thus if $\sigma_T \propto \sqrt{\tau}$, we obtain $\tau_{\rm corr} \propto 1/\tau$.
