# Supplementary material for: Transcription Fluctuation Effects on Biochemical Oscillations
Source: PLoS One. 2013 Apr 12;8(4):e60938. doi: 10.1371/journal.pone.0060938 (PMC3625213; doi:10.1371/journal.pone.0060938)
Supplement: Supporting Information S2 — Supplementary Material. (PDF) [file pone.0060938.s002.pdf]

**Supplementary material to**
**"Transcription fluctuation effects on biochemical oscillations"**
**by Ryota Nishino, Takahiro Sakaue, and Hiizu Nakanishi**

In this supplementary material, we present data to demonstrate the $\sqrt{\tau}$-scaling of the distribution width and the multiple gene effects more systematically.

## S1 $\sqrt{\tau}$-scaling of the distribution width

In the text, we show only three sets of data for the distributions of the period and the peak value of $[P_C]$ in order to demonstrate the $\sqrt{\tau}$ scaling of the distribution width. We examined this scaling more systematically.

In Fig.S1, the ratios of the standard deviation to the average are plotted against $\tau$ in the logarithmic scale. The dashed lines denote the fitting lines with the slope 0.5, which shows the $\sqrt{\tau}$ scaling of the distribution width of the oscillation parameters in the small $\tau$ region.

This can be understood naturally; The on/off frequency of the gene is proportional to $1/\tau$, thus the fluctuation in the total on/off-time length scales with $\sqrt{\tau}$, from which we expect that the distribution width of the oscillation parameters scales with $\sqrt{\tau}$ because the response in fluctuation should be linear in the small input fluctuation limit.

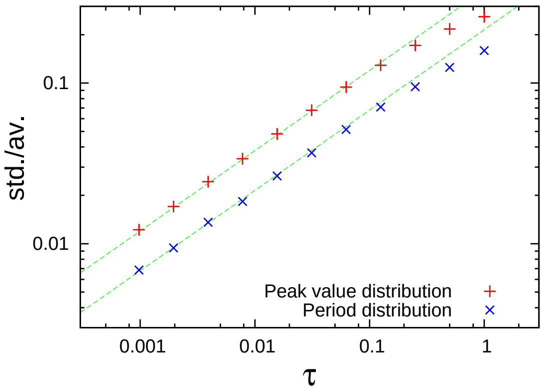


Figure S1: Scaling of the distribution width by $\tau$. The ratio of the standard deviation to the average is plotted against $\tau$ in the logarithmic scale for the peak value of $[P_C]$ and the period distributions. The dashed lines are the lines with the slope 0.5 fitted to each data set.

## S2 Multiple gene effects

In the paper, we only consider the case where the copy number of the gene is one. Here, we present the analysis for the case where the copy number of the gene is $G_0$. The transition rate for each reaction is given by Table S1, where $G$ is the number of active genes. Note that the transition rate for the reaction 1 is scaled by $1/G_0$ in order to give the same rate equation as before in the limit of $\tau \to 0$ and $\Omega \to \infty$.

In the $\tau \to 0$ limit, the time dependent average of $G$ denoted by $G_{\rm Av}(t)$ is given by

$$G_{\rm Av}(t) = G_0 \frac{1}{1 + ([{\rm P_N}]/K_I)^n}, \tag{S.1}$$

which gives the same rate equations for the concentrations if the transition rate for the reaction 1 is scales by $1/G_0$.

To see the copy number effects, we performed Monte Carlo simulations for $G_0 = 1$ and 2 with some values of $\tau$ in the $\Omega \to \infty$ limit. Fig. S2 shows the distributions for the period and the peak values of $[{\rm P_C}]$. One can see the distribution for $G_0 = 2$ with $\tau = 0.1$ h agrees with that for $G_0 = 1$ with $\tau = 0.05$ h quite well for both of the distributions.

Actually, this can be understood in a simple way; For the copy number $G_0 = 2$, the fluctuations in the activity of the two genes cancel each other. This cancellation should be comparable to the fluctuation cancellation in the system with $G_0 = 1$ and the half time scale because the gene activity switches between on and off twice as fast.

| no. | reaction | transition rate |
|---|---|---|
| a | $\begin{matrix} G \\ P_N \end{matrix} \longrightarrow \begin{matrix} G-1 \\ P_N - n \end{matrix}$ | $\frac{1}{\tau}\left(\frac{P_N}{K_I\Omega}\right)^n G$ |
| b | $\begin{matrix} G \\ P_N \end{matrix} \longrightarrow \begin{matrix} G+1 \\ P_N + n \end{matrix}$ | $\frac{1}{\tau}(G_0 - G)$ |
| 1 | $M \longrightarrow M+1$ | $v_s\Omega \frac{G}{G_0}$ |
| 2 | $M \longrightarrow M-1$ | $v_m\Omega \frac{M/\Omega}{K_m + M/\Omega}$ |
| 3 | $P_C \longrightarrow P_C + 1$ | $k_s M$ |
| 4 | $P_C \longrightarrow P_C - 1$ | $v_d\Omega \frac{P_C/\Omega}{K_d + P_C/\Omega}$ |
| 5 | $\begin{matrix} P_C \\ P_N \end{matrix} \longrightarrow \begin{matrix} P_C - 1 \\ P_N + 1 \end{matrix}$ | $k_1 P_C$ |
| 6 | $\begin{matrix} P_C \\ P_N \end{matrix} \longrightarrow \begin{matrix} P_C + 1 \\ P_N - 1 \end{matrix}$ | $k_2 P_N$ |

Table S1: Reaction table for a simplified circadian system in the case where the copy number of the gene is $G_0$.

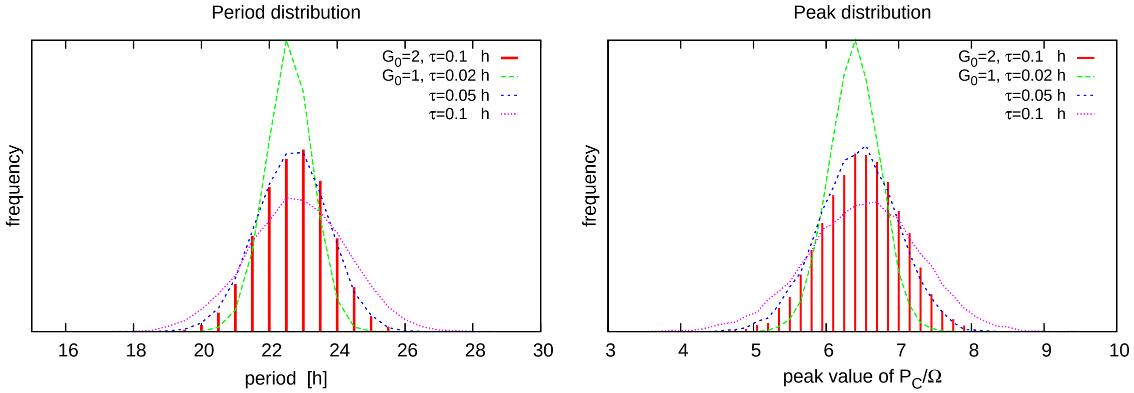


Figure S2: Period and $[\mathrm{P_C}]$ peak value distributions for $G_0 = 1$ and 2 with various values of $\tau$ in the $\Omega \to \infty$ limit. The other parameters are the same with those in the text: $n = 4$, $v_s = 0.5$ nM h$^{-1}$, $K_I = 2.0$ nM, $v_m = 0.3$ nM h$^{-1}$, $K_m = 0.2$ nM, $k_s = 2.0$ h$^{-1}$, $v_d = 1.5$ nM h$^{-1}$, $K_d = 0.1$ nM, $k_1 = 0.2$ h$^{-1}$, $k_2 = 0.2$ h$^{-1}$.
