# Supplementary material for: Transcription Fluctuation Effects on Biochemical Oscillations
Source: PLoS One. 2013 Apr 12;8(4):e60938. doi: 10.1371/journal.pone.0060938 (PMC3625213; doi:10.1371/journal.pone.0060938)
Supplement: Supporting Information S3 — Footnotes. (PDF) [file pone.0060938.s003.pdf]

Transcription fluctuation effects on biochemical oscillations

Ryota Nishino, Takahiro Sakaue, Hiizu Nakanishi

Foot notes

1. In their work[12], the reaction rates for the gene processes a_i and d_i $(i = 1 \sim 4)$ are taken to be proportional to Ω as shown in Table 2 of Supporting information for ref.[12].

2. Ref.[12] examined the case of the sequential $\mathrm{P_N}$ bindings, but there are other possible decompositions of the process such as that some dimers or oligomers bind to the gene after being formed in the nucleus.

3. Ref.[12] used Ω as a scaling parameter in the places where we use $1/\tau$.
