# Supplementary material for: Correction: Transcription Fluctuation Effects on Biochemical Oscillations
Source: PLoS One. 2014 Apr 9;9(4):e95119. doi: 10.1371/journal.pone.0095119 (PMC3981862; doi:10.1371/journal.pone.0095119)
Supplement: File S1 — Originally published, uncorrected article. (PDF) [file pone.0095119.s001.pdf]

OPEN ACCESS Freely available online PLOS ONE

Transcription Fluctuation Effects on Biochemical Oscillations

Ryota Nishino, Takahiro Sakaue, Hiizu Nakanishi*

Department of Physics, Kyushu University, Fukuoka, Japan

Abstract

Some biochemical systems show oscillation. They often consist of feedback loops with repressive transcription regulation. Such biochemical systems have distinctive characteristics in comparison with ordinary chemical systems: i) numbers of molecules involved are small, ii) there are typically only a couple of genes in a cell with a finite regulation time. Due to the fluctuations caused by these features, the system behavior can be quite different from the one by deterministic rate equations, because the rate equations ignore molecular fluctuations and thus are exact only in the infinite molecular number limit. The molecular fluctuations on a free-running circadian system have been studied by Gonze et al. (2002) by introducing a scale parameter Ω for the system size. They consider, however, only the first effect, assuming that the gene process is fast enough for the second effect to be ignored, but this has not been examined systematically yet. Here we study fluctuation effects due to the finite gene regulation time by introducing a new scale parameter τ, which we take as the unbinding time of a nuclear protein from the gene. We focus on the case where the fluctuations due to small molecular numbers are negligible. In simulations on the same system studied by Gonze et al., we find the system is unexpectedly sensitive to the fluctuation in the transcription regulation; the period of oscillation fluctuates about 30 min even when the regulation time scale τ is around 30 s, that is even smaller than 1/1000 of its circadian period. We also demonstrate that the distribution width for the oscillation period and amplitude scales with $\sqrt{\tau}$, and the correlation time scales with $1/\tau$ in the small τ regime. The relative fluctuations for the period are about half of that for the amplitude, namely, the periodicity is more stable than the amplitude.

Citation: Nishino R, Sakaue T, Nakanishi H (2013) Transcription Fluctuation Effects on Biochemical Oscillations. PLoS ONE 8(4): e60938. doi:10.1371/journal.pone.0060938

Editor: Jordi Garcia-Ojalvo, Universitat Politecnica de Catalunya, Spain

Received October 14, 2012; **Accepted** March 4, 2013; **Published** April 12, 2013

Copyright: © 2013 Nishino et al. This is an open-access article distributed under the terms of the Creative Commons Attribution License, which permits unrestricted use, distribution, and reproduction in any medium, provided the original author and source are credited.

Funding: The funding was provided by Kyushu University. The funders had no role in study design, data collection and analysis, decision to publish, or preparation of the manuscript.

Competing Interests: The authors have declared that no competing interests exist.

* E-mail: nakanisi@phys.kyushu-u.ac.jp

Introduction

One of the outstanding features in biological systems is that the systems often operate on surprisingly small numbers of active molecules, yet they seem to work quite reliably. This is especially intriguing in the case where the chemical reaction system involves a gene transcription because there are typically only a couple of genes in a cell, and their stochasticity is known to produce significant fluctuations [1–5].

One example is a circadian system, which shows a rhythmic behavior of approximately 24-hour periodicity. It is a universal feature of biological systems and known to be very accurate and robust against external and internal perturbations [6,7]. Its biochemical mechanisms have been proposed in several systems [8–10], and most of them are based on a time-delayed negative feedback loop of a biochemical reaction network which includes transcription regulations. Some of the protein molecules are expected to be very small in number, and the number of each gene is typically of the order of one in a cell and does not scale with the cell size, thus it is surprising that the circadian system is capable of maintaining its extraordinary regularity especially in the case of a single cell organism [11].

The effects of molecular fluctuations on the circadian system has been studied by [12,13] by Monte Carlo simulations using the Gillespie method [14,15] with the scale parameter Ω for the molecular numbers. By simulating the system with various values of the scaling parameter Ω, they demonstrated that the system shows reasonably coherent oscillation as long as the system contains more than several tens of mRNA, thus concluded that their system are fairly robust against molecular fluctuations.

They examined the system rather systematically based upon a standard method to study the stochastic nature of chemical reactions [16] by scaling the reaction rates in the way to keep the rate equation unchanged. However, there is an ambiguity in the treatment of the gene regulation process because the number of genes should not scale with other protein numbers. They scaled the reaction rates proportional to Ω for the gene processes (see 1 in Supporting Information S3), namely, they made the gene regulation times infinitely fast in the large Ω limit, thus the system dynamics reduce to the one described by the corresponding rate equations without the gene processes [12]. This could be justified only when the gene processes are so fast that they do not cause significant fluctuation on the system behavior. In fact, it is not reasonable to assume that the time scale of the gene regulation depends upon the scale parameter Ω, if you think of it as the cell volume, because the time scale with which a regulatory protein binds to the operator site is determined by the protein concentration, and the time scale with which the protein unbinds is determined by its binding energy.

In this work, in order to analyze fluctuations from the two distinct origins separately, we introduce a new scale parameter τ in addition to Ω. The scale parameter τ scales the binding/unbinding time of the gene regulatory protein. Thus, these two parameters, τ and Ω, control the two distinct fluctuation sources that exist in the biological systems, namely, Ω controls the fluctuations due to the finite molecular numbers while τ controls the fluctuations due to the finite gene regulation times. We perform the Monte Carlo simulations on the same system as the one studied by [13], focusing on the latter effect, and demonstrate that significant fluctuation can arise from the stochasticity in the gene process alone. We also examine how the fluctuation scales with τ.

Model

The model we study is the simplest version of core model for a circadian system that consists of a gene G, mRNA M, cytosolic protein P_C, and nuclear protein P_N (Fig. 1). The biochemical reactions for these elements are given by

$$\mathrm{G} + n\mathrm{P_N} \rightleftarrows \mathrm{GP_{Nn}}, \quad (1)$$

$$\mathrm{G} \rightarrow \mathrm{G} + \mathrm{M}, \quad (2)$$

$$\mathrm{M} \rightarrow \times, \quad (3)$$

$$\mathrm{M} \rightarrow \mathrm{M} + \mathrm{P_C}, \quad (4)$$

$$\mathrm{P_C} \rightarrow \times, \quad (5)$$

$$\mathrm{P_C} \rightleftarrows \mathrm{P_N}. \quad (6)$$

The parameter n is the number of nuclear proteins P_N that bind to suppress the gene, i.e. Hill coefficient for the gene activity; we adopt $n = 4$ for most of the calculation as [12,13]. The reaction (1) may be decomposed further into several chemical steps(see 2 in Supporting Information S3), but we focus on the simple case where there exists a rate limiting process that dominates the gene process, and the overall process can be effectively represented by the reaction (1) with the Hill coefficient n.

Now, we introduce the two scaling parameter Ω and τ; Ω scales the reaction rates so that the numbers of mRNA and the proteins become proportional to it, and τ scales the binding/unbinding time of the nuclear protein to the gene operator site. The transition rates for each reaction are listed in Table 1, where we define the variables G, M, P_C, and P_N to represent the numbers of active genes, mRNA, cytosolic proteins, and the nuclear proteins in a single cell, respectively. The gene variable G takes either 1 or 0 values, depending upon the active state (G) or the inactive state ($\mathrm{GP_{Nn}}$), respectively. Note that we employ Michaelis-Menten enzymatic reactions for the degradation processes. The first two reaction rates in Table 1 are for the gene regulation and proportional to $1/\tau$, but do not scale with Ω because we assume only one gene in a cell (see 3 in Supporting Information S3). The ratio of the binding and the unbinding times is determined in the way that the corresponding average behavior described by the rate equation remains the same with the original system in the small τ limit. On the other hand, the gene transcription activity in the third reaction is scaled as $v_s\Omega$ in order that the numbers of mRNA and the proteins should be proportional to Ω.

If we naively write down differential equations for the time evolution, ignoring the fact that the variables are integers, we would have

$$\tau \frac{dG}{dt} = (1-G) - \left(\frac{P_N/\Omega}{K_I}\right)^n G, \quad (7)$$

$$\frac{dM}{dt} = v_s\Omega G - v_m\Omega \frac{M}{K_m\Omega + M}, \quad (8)$$

$$\frac{dP_C}{dt} = k_s M - v_d\Omega \frac{P_C}{K_d\Omega + P_C} - k_1 P_C + k_2 P_N, \quad (9)$$

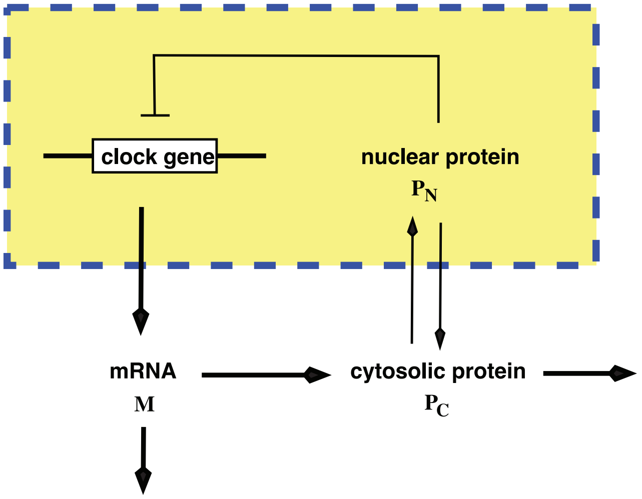

Figure 1. Simplified core model for a circadian system.
doi:10.1371/journal.pone.0060938.g001

Table 1. Reaction table for a simplified circadian system.

no.	reaction	transition rate
a	$G=0 G=1 \rightarrow$	$\frac{1}{\tau}\left(\frac{P_N}{K_I\Omega}\right)^n G$
	$P_N - nP_N$	
b	$G=1 G=0 \rightarrow$	$\frac{1}{\tau}(1-G)$
	$P_N + nP_N$	
1	$M+1 M \rightarrow$	$v_s\Omega G$
2	$M-1 M \rightarrow$	$v_m\Omega\frac{M/\Omega}{K_m+M/\Omega}$
3	$P_C+1 P_C \rightarrow$	$k_s M$
4	$P_C-1 P_C \rightarrow$	$v_d\Omega\frac{P_C/\Omega}{K_d+P_C/\Omega}$
5	$P_C-1 P_C \rightarrow$	$k_1 P_C$
	$P_N+1 P_N$	
6	$P_C+1 P_C \rightarrow$	$k_2 P_N$
	$P_N-1 P_N$	

doi:10.1371/journal.pone.0060938.t001

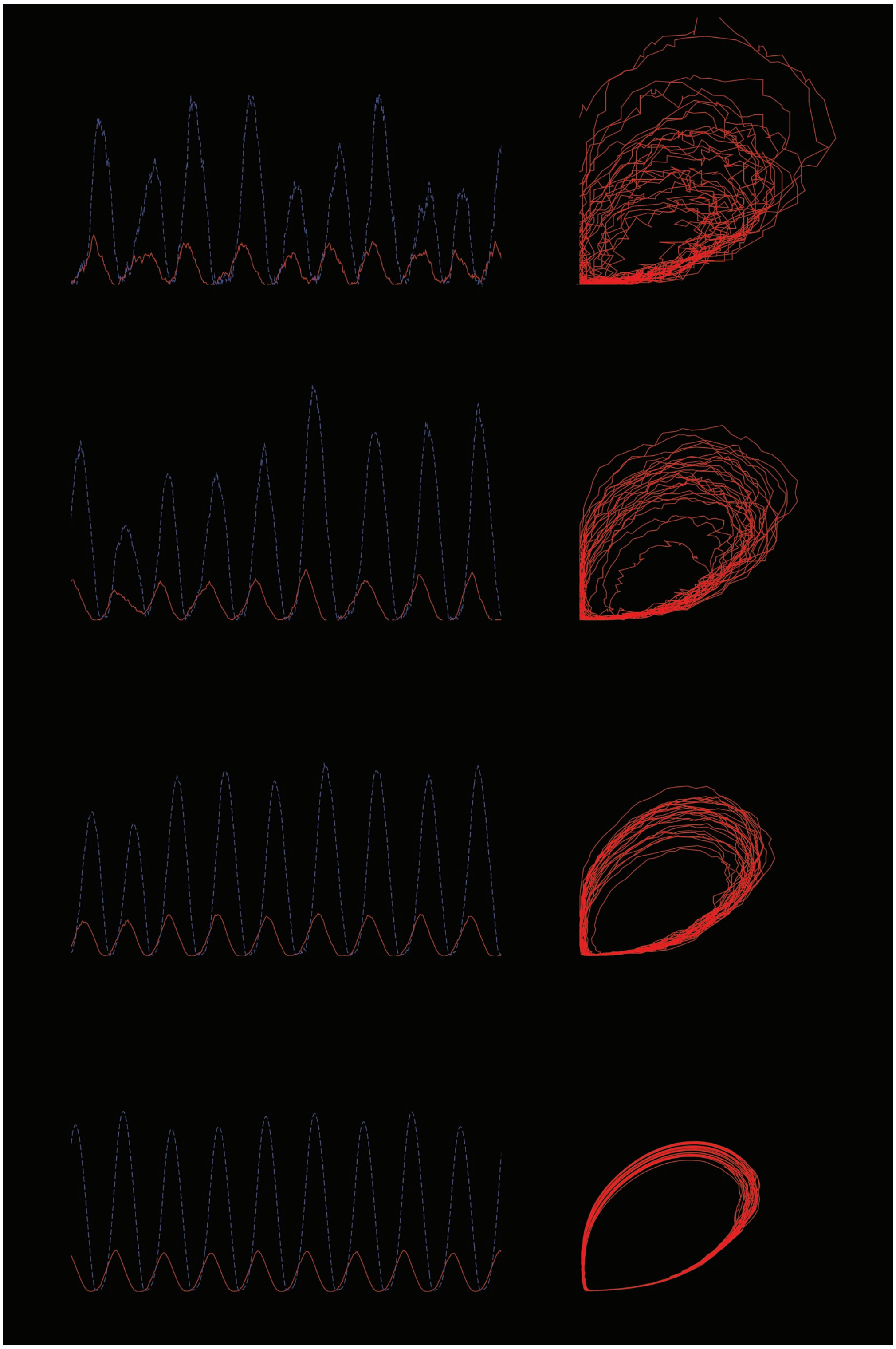

Figure 2. Oscillatory behaviors of the concentrations of mRNA and the cytosolic protein for various values of Ω with $\tau = 0.01$ h. The plots in the left column show the time variation and those in the right column are the projections of the trajectories in the $\mathrm{M}/\Omega - \mathrm{P_C}/\Omega$ plane. We employ the same reaction parameters with those in Gonze et al.: $n = 4$, $v_s = 0.5$ nM h^{-1}, $K_I = 2.0$ nM, $v_m = 0.3$ nM h^{-1}, $K_m = 0.2$ nM, $k_s = 2.0$ h^{-1}, $v_d = 1.5$ nM h^{-1}, $K_d = 0.1$ nM, $k_1 = 0.2$ h^{-1}, $k_2 = 0.2$ h^{-1}.
doi:10.1371/journal.pone.0060938.g002

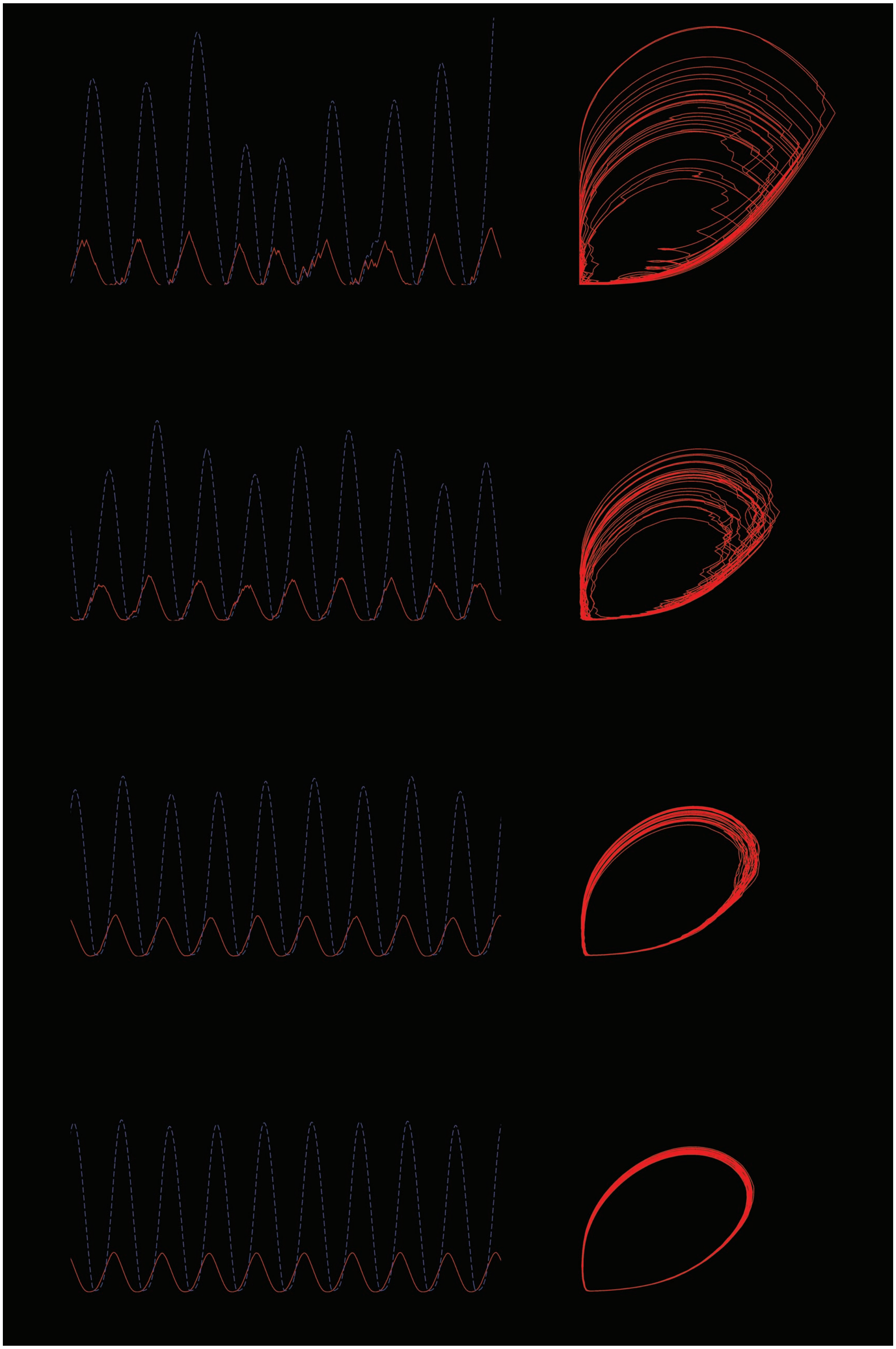

Figure 3. Oscillatory behaviors of the concentrations of mRNA and the cytosolic protein for various values of τ with $\Omega = \infty$. The parameters are the same with those in Fig. 2.
doi:10.1371/journal.pone.0060938.g003

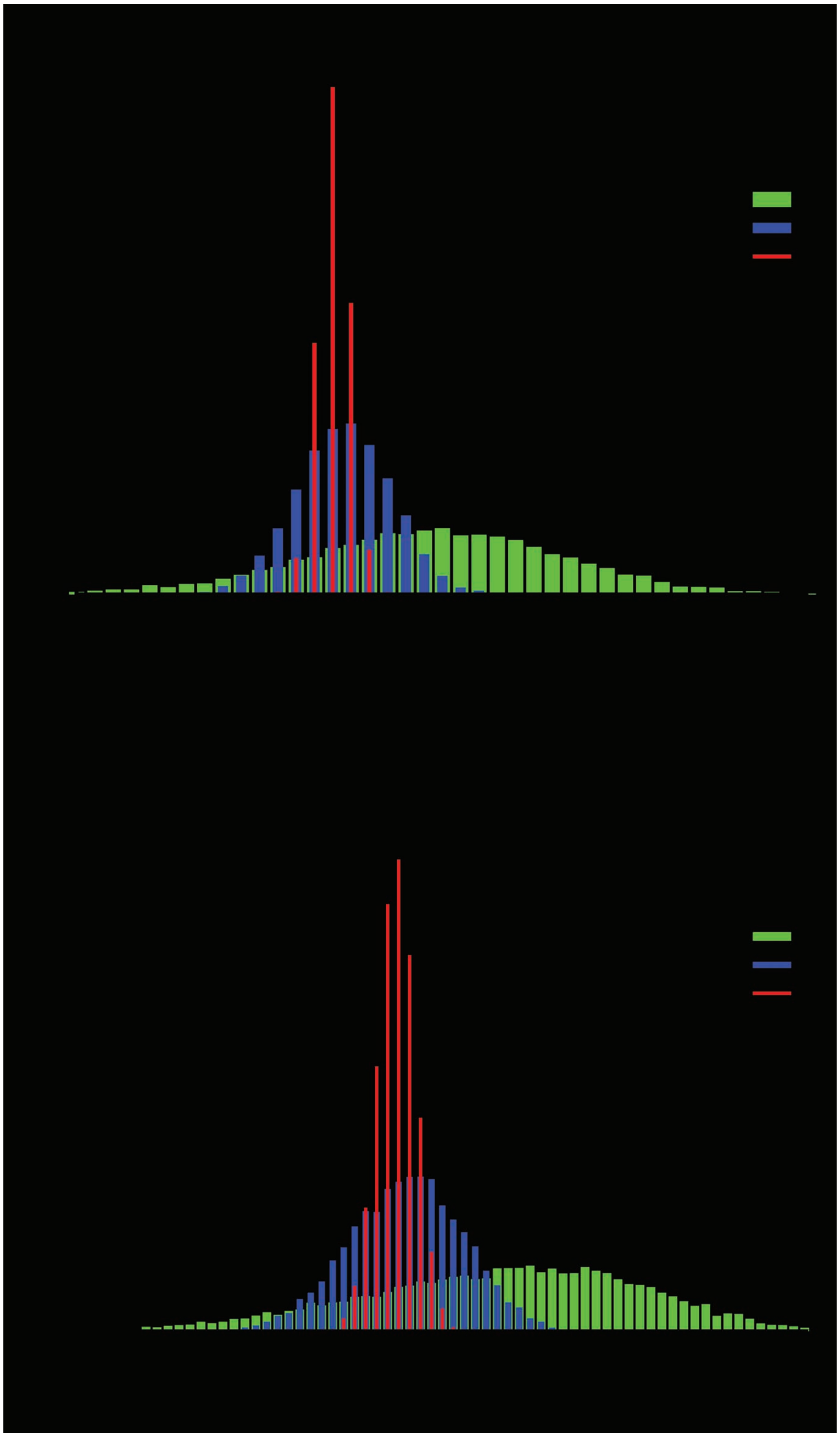

Figure 4. Distributions for (a) the period (i.e. peak-to-peak interval) and (b) the peak value of the cytosolic protein variation for $\tau = 1.0$, 0.1, 0.01 h with $\Omega = \infty$. The averages and the standard deviations are tabulated in Table 2, from which one can see that the width of the distribution scales roughly as $\sqrt{\tau}$.
doi:10.1371/journal.pone.0060938.g004

$$\frac{dP_N}{dt} = k_1 P_C - k_2 P_N \tag{10}$$

$$+\frac{n}{\tau}\left[(1-G)-\left(\frac{P_N/\Omega}{K_I}\right)^n G\right]. \tag{11}$$

Table 2. The averages and the standard deviations for the period and the peak value distributions for the cytosolic protein concentration shown in Fig. 4.

τ (h)		1	0.1	0.01
	av.	24.6	22.3	22.0
period (h)	std.	3.92	1.32	0.45
	std./av.	0.16	0.059	0.020
	av.	7.82	6.50	6.37
peak value (nM)	std.	1.87	0.79	0.26
	std./av.	0.24	0.12	0.041

The ratios of the standard deviation to the average for the peak value distributions are about twice as large as those for the period distributions.
doi:10.1371/journal.pone.0060938.t002

For ordinary chemical reactions without a gene transcription process, the stochastic dynamics should be well described by such equations for the large Ω case, where the numbers of molecules are large. However, in the present system, the number of gene is one and does not scale with Ω, thus the stochastic nature remains even in the case of the infinite Ω as long as τ is finite.

Large Ω Limit

Supposing the scale parameter Ω as a cell volume, we define the "concentrations" of mRNA and the proteins as,

$$[\mathrm{M}] \equiv \frac{M}{\Omega}, \quad [\mathrm{P_C}] \equiv \frac{P_C}{\Omega}, \quad [\mathrm{P_N}] \equiv \frac{P_N}{\Omega}, \tag{12}$$

and write down the rate equations for them as

$$\frac{d[\mathrm{M}]}{dt} = v_s G - v_m \frac{[\mathrm{M}]}{K_m + [\mathrm{M}]}, \tag{13}$$

$$\frac{d[\mathrm{P_C}]}{dt} = k_s[\mathrm{M}] - v_d \frac{[\mathrm{P_C}]}{K_d + [\mathrm{P_C}]} - k_1[\mathrm{P_C}] + k_2[\mathrm{P_N}], \tag{14}$$

$$\frac{d[\mathrm{P_N}]}{dt} = k_1[\mathrm{P_C}] - k_2[\mathrm{P_N}], \tag{15}$$

where we have ignored the term of the order of n/Ω in Eq.(15).

For ordinary chemical reactions, we expect that the deterministic dynamics represented by the rate equations would describe the system accurately in the large Ω limit, because the effect of molecular fluctuation becomes negligible. However, for the present case, the system remains stochastic even in the large Ω limit because the variable G remains stochastic.

Small τ Limit

In the case where τ is much smaller than any other time scales in the system, the system reduces to the one studied by [13]. This can be seen by introducing the time dependent average value of G, denoted by $G_{\mathrm{Av}}(t)$, i.e. the time average of G over the longer time scale than τ but shorter than other time scales in the system. Its value is given by the condition that the first two reactions in Table 1 are equilibrated,

$$G_{\mathrm{Av}}(t) = \frac{1}{1 + ([\mathrm{P_N}]/K_I)^n}. \tag{16}$$

Then the system dynamics are given by the stochastic dynamics of reaction $1 \sim 6$ in Table 1 with G replaced by G_{Av}.

If we take the large Ω limit on top of this, we obtain the deterministic rate equations given by Eqs.(13)~(15) with G being replaced by $G_{\mathrm{Av}}(t)$ of Eq.(16).

Simulations and Results

In order to examine the effect of gene fluctuations, we have performed numerical simulations for various values of τ and Ω. We examine two cases: (i) the case where both τ and Ω are finite, and (ii) the case where τ is finite but in the large Ω limit. In the first case, the fully stochastic dynamics are given by Table 1; for these we employ the Gillespie method [14,15]. In the second case, the concentrations [M], [$\mathrm{P_C}$], and [$\mathrm{P_N}$] follow the deterministic dynamics while the gene process remains stochastic. In this case, we integrate the rate equations (13)~(15) using Runge-Kutta method, but at every time step of the length Δt, the gene variable G is subject to a trial for change according to the probability $w\Delta t$ under Poisson process with w being the transition rate given in the first two processes in Table 1.

Figure 2 shows the system behaviors for various values of Ω with $\tau = 0.01$h. The other reaction parameters are the same as those used by [13]. The plots in the left column are the time variations of the concentrations of mRNA (solid lines) and the cytosolic protein (dashed lines), and those in the right column show the oscillation trajectories projected on the $M/\Omega - P_C/\Omega$ plane of the phase space. The fluctuation decreases as Ω increases, but it remains finite even in the infinite Ω case because of the fluctuation from the gene activity.

In order to see the effect of gene stochasticity, we examine the case for various values of τ in the large Ω limit (Fig. 3). The fluctuation decreases on decreasing τ as in the case of increasing Ω. The trajectories are smoother in comparison with the previous case because the stochasticity is limited to the gene activity. One can see that the fluctuation is evident even in the case $\tau = 0.01$h, where the ratio τ to the period (~24 h) is as small as 0.5×10^{-3}.

Figure 4 shows the period (i.e. the peak-to-peak interval) distributions and the peak value distributions of [$\mathrm{P_C}$] for $\tau = 1.0$, 0.1, and 0.01 h. The averages and the standard deviations for the distributions are tabulated in Table 2. Both of the distributions become narrower for the smaller value of τ approximately proportional to $\sqrt{\tau}$, but the standard deviation of the period distribution is still about a half hour even for the case of $\tau = 0.01$ h. It should be noted that the ratios of the standard deviation to the average for the peak value distributions are about twice as large as those for the period distributions. More systematic data are presented in Supporting Information S2 to show the $\sqrt{\tau}$ scaling and the ratio of the two distribution widths. As for the averages of the distributions, they shift toward larger values for larger τ. This is because the system tends to make larger loops of oscillation when P_N binding is delayed, therefore, the gene activity is prolonged due to a small binding constant.

The time correlation function $C(t)$ of the nuclear protein concentration [$\mathrm{P_N}$(t)] is defined as

$$C(t) = \frac{1}{T}\int_0^T \Delta[\mathrm{P_N}(t'+t)]\,\Delta[\mathrm{P_N}(t')]\,dt', \tag{17}$$

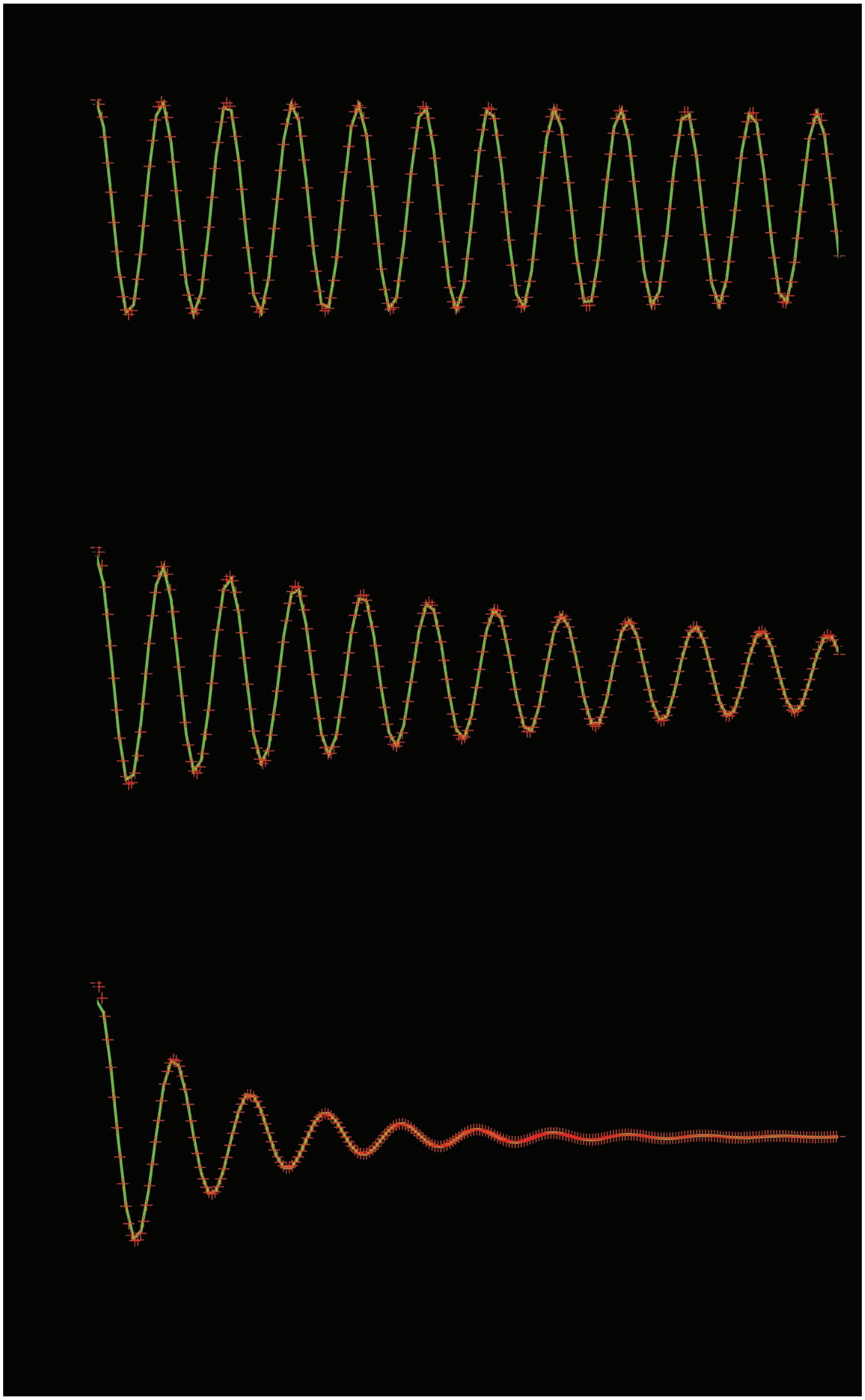

Figure 5. The time correlation functions for the nuclear protein concentration for the regulation time $\tau = 0.01$, 0.1, 1 h with $\Omega = \infty$. The (green) lines shows the fitting curves of the form $A\cos(\omega_0 t + \theta_0)e^{-t/\tau_{\mathrm{corr}}}$. The fitted values of τ_{corr} are shown on the plots. The other parameters are the same with those in Fig. 2.

doi:10.1371/journal.pone.0060938.g005

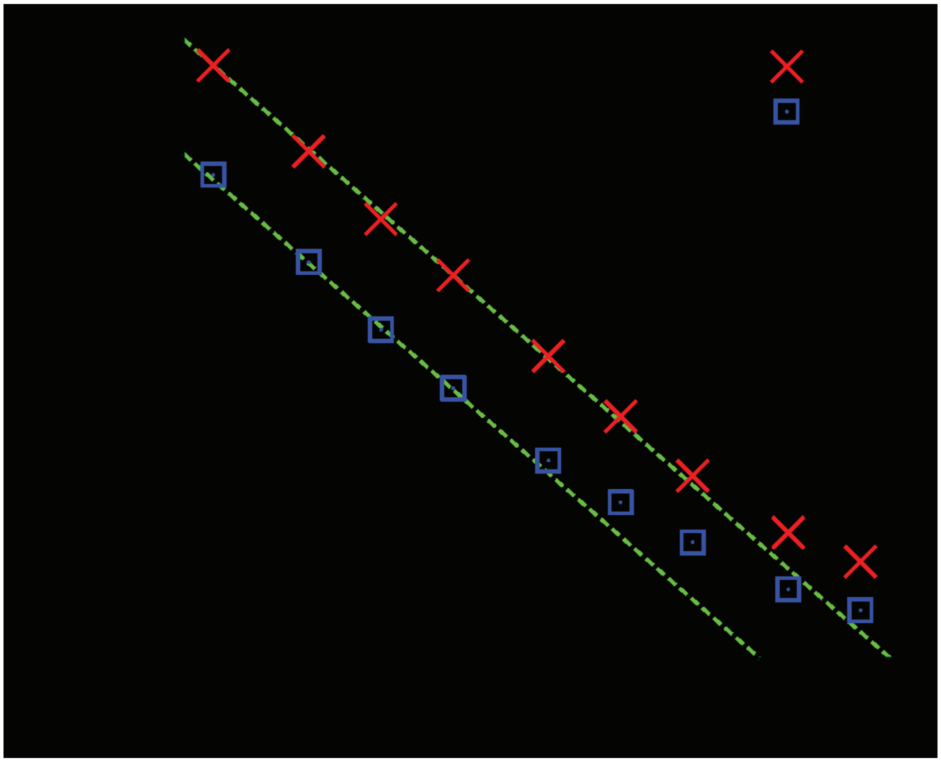

Figure 6. The regulation time τ dependences of the correlation time τ_{corr} for $n=1$ and 4 with $\Omega=\infty$ in the logarithmic scale. The (green) dashed lines shows the fitted lines proportional to τ^{-1}.
doi:10.1371/journal.pone.0060938.g006

where $\Delta[P_N(t)]$ represents the deviation from the average,

$$\Delta[P_N(t)] \equiv [P_N(t)] - \frac{1}{T}\int_0^T [P_N(t')]\,dt' \qquad (18)$$

with T being the time length of the whole simulation. In Fig. 5, the correlation functions are plotted and fitted to the form of damped oscillation

$$A\,\cos\,(\omega_0 t + \theta_0)\,e^{-t/\tau_{corr}} \qquad (19)$$

to estimate the correlation time τ_{corr}. Figure 6 shows the τ dependence of the correlation time τ_{corr} in the logarithmic scale. It shows the scaling

$$\tau_{corr} \sim \frac{1}{\tau} \qquad (20)$$

in the small τ regime, and the longer correlation time in the $n=4$ case than in the $n=1$ case. One may notice that the correlation time for $\tau=0.01$ h is quite long, i.e. $\tau_{corr} \approx 2000$ h for the $n=4$ case, even though the period fluctuations are substantial as can be seen in Fig. 4 (See Supporting Information S1).

The scaling of τ_{corr} given by Eq.(20) can be understood as the phase diffusion when the standard deviations of the period distributions scales as $\sqrt{\tau}$ as shown in Fig. 4.

Discussion

We have examined the effects of molecular fluctuations in a biological system on a simplified model of a circadian rhythm system, where there are two types of fluctuation sources: (i) small numbers of molecules involved and (ii) finite time scale of the gene regulation. The first effect has been studied by [13], assuming that the gene regulation time scale is infinitesimal. In the present work, we focus on the second effect, i.e., in the case where the molecular numbers are large enough that the fluctuation due to the first effect is negligible.

We have developed a method to study this effect systematically by introducing a new parameter τ to scale the gene regulation times. We set τ to be the unbinding time of the transcription factor, keeping the ratio of the binding to the unbinding rate constant. We performed numerical simulations for various values of τ without an external entrainment of the 24-hour period. As τ decreases, the oscillation appears more deterministic; the width of the distributions for the oscillation periods and the peak values scales with $\sqrt{\tau}$ and the correlation time for the correlations function scales with $1/\tau$. We have found that the system is very sensitive to such fluctuation, and demonstrated that the oscillation period fluctuates by about 30 min even for very small $\tau=0.01$ h ≈ 30 s in comparison with its period around 22 h. For the present parameter set, the nuclear protein concentration P_N/Ω oscillates in the range 0~5 nM, therefore, the value of $\tau \approx 30$ s for the unbinding time gives about 1 s for the binding time. These estimates may be tested with experimental data.

The 30 minutes period fluctuation is large for a circadian system. This sensitivity to the fluctuation in the gene regulation is an interesting feature of the present simplified model. Multiple feedback loops with several phosphorylation steps found in actual

biological systems have been discussed in the context of stabilization mechanism of the oscillation [17,18]; Such redundancy in the systems may also provide reduction mechanism of this sensitivity. Such possibility can be studied by extending the present method.

The correlation function for the protein oscillation fits to the damped sinusoidal function very well, and the estimated correlation time $\tau_{\rm corr}$ scales as $1/\tau$ in the small τ regime. Such decay in the correlation function is caused by the phase diffusion due to fluctuations. We estimate $\tau_{\rm corr}$ for the Hill coefficient for the gene regulation $n=4$ and 1, and found that $\tau_{\rm corr}$'s for $n=4$ are about 5 times larger than those for $n=1$; the fluctuation effect is suppressed by the larger value of the Hill coefficient by the cooperativity effects as in the case of [12,13].

It is also interesting to find that the relative fluctuations for the peak values are twice as large as those for the periods, namely, the period is more stable than the amplitude. This may be a reason why the correlation time is quite long in spite of apparent fluctuations in the oscillation.

In the present work, we study only the case where the copy number of the gene is 1. However, there are typically a couple of genes in a cell. In the case of multiple genes in a cell, the fluctuation in each gene cancels each other, therefore overall fluctuation will be reduced. We confirmed by simulation that the fluctuation for a two-gene system with τ is almost the same as that for a single gene system with $\tau/2$. This is because the fluctuation cancellation by two genes should be comparable with that by one gene that switches twice as fast. Simulation data are presented in Supporting Information S2.

The fluctuation indicated by our simulations may be compared with previous experimental observations. Although circadian clocks are very accurate as a system, large fluctuations have been observed in the oscillation of individual cells of fibroblasts [19] and cyanobacteria [20] when they oscillate independently. For both cases, it is reported that the fluctuations are much larger for the amplitude than those for the period. In the latter case [20], the correlation time is estimated as long as 166 ± 100 days in spite of apparent large fluctuations in the amplitude. Such a long correlation time corresponds to our case of the gene regulation time scale $\tau=0.01$ h, which gives the $\tau_{\rm corr}\approx1950$ h.

Very little fluctuation is usually observed in circadian systems; fluctuation in the period is typically less than 10 minutes [21], which is even smaller than the fluctuation of 30 minutes that we obtained for the case $\tau=0.01$ h. There are some possible mechanisms to suppress molecular fluctuations. (i) Cooperativity among cells: The present system models a single cell behavior, but for the case of multicellular organisms, the cooperativity among cells may exist and that should reduce the fluctuation in each cell. Actually, variability in each cell is much larger than that of a whole system in the case of multicellular organisms [22–25]. (ii) Multiple feedback loops: Our model is a simplified core model for a circadian system and consists of a single negative feedback loop. However, it has been known that circadian systems typically consist of multiple feedback loops [26–29], which could be designed in the way to compensate the fluctuations in one loop by the other. (iii) Chemical oscillation without gene control: In the case of cyanobacteria, it has been proposed that the circadian system consists of proteins only and does not involve a gene expression [30]. In such a system, the fluctuation discussed in this work does not exist.

Other than circadian systems, there are some oscillations observed in biology such as Hes1 oscillation during somite segmentation [31], p53 oscillation after DNA damage by gamma irradiation [32], or oscillations in artificially constructed systems [33–35]. In these systems, the fluctuations are much more profound than circadian systems, and part of the fluctuations should come from the gene regulatory processes, for which the present analysis is applicable.

In summary, we have developed a theoretical tool to study the molecular fluctuation due to the finite transcription regulation time, and have demonstrated that a simplified core model of circadian system is sensitive to such fluctuation. Our method can be extended to study a more realistic system and can be utilized to clarify biological significance of a detailed design of circadian system in terms of stability against the molecular fluctuation.

Supporting Information

Supporting Information S1 Appendix.
(PDF)

Supporting Information S2 Supplementary Material.
(PDF)

Supporting Information S3 Footnotes.
(PDF)

Acknowledgments

The authors would like to acknowledge Dr. Hiroshi Ito for informative discussions.

Author Contributions

Analyzed the data: TS HN. Wrote the paper: HN. Performed the numerical simulations: RN HN.

References

1. Elowitz MB, Levine AJ, Siggia ED, Swain PS (2002) Stochastic gene expression in a single cell. Science 297: 1183–1186.
2. Kærn M, Elston TC, Blake WJ, Collins JJ (2005) Stochasticity in gene expression: from theories to phenotypes. Nature Reviews 6: 451–464.
3. Raser JM, O'Shea EK (2005) Noise in gene expression: Origins, consequences, and control. Science 309: 2010–2013.
4. Shahrezaei V, Swain PS (2008) The stochastic nature of biochemical networks. Curr Opin Biotech- nol 19: 369–374.
5. Raj A, van Oudenaarden A (2008) Nature, nurture, or chance: Stochastic gene expression and its consequences. Cell 135: 216–226.
6. Dunlap JC (1999) Molecular bases for circadian clocks. Cell 96: 271–290.
7. Young MW (2000) Life's 24-hour clock: molecular control of circadian rhythms in animal cells. Trends Biochem Sci 25: 601–606.
8. Goldbeter A (1995) A model for circadian oscillations in the drosophila period protein (per). Proc R Soc Lond B 261: 319–324.
9. Goldbeter A (1996) Biochemical Oscillations and Cellular Rhythms: The Molecular Bases of Periodic and Chaotic Behaviour. Cambridge, UK: Cambridge University Press.
10. Leloup JC, Gonze D, Goldbeter A (1999) Limit cycle models for circadian rhythms based on transcriptional regulation in drosophila and neurospora. J Biol Rhythms 14: 433–448.
11. Barkai N, Leibler S (2000) Biological rhythms: Circadian clocks limited by noise. Nature 403: 267–268.
12. Gonze D, Halloy J, Goldbeter A (2002) Robustness of circadian rhythms with respect to molecular noise. PNAS 99: 673–678.
13. Gonze D, Halloy J, Gaspard P (2002) Biochemical clocks and molecular noise: Theoretical study of robustness factors. J Chem Phys 116: 10997–11010.
14. Gillespie DT (1976) A general method for numerically simulating the stochastic time evolution of coupled chemical reactions. J Comput Phys 22: 403–434.
15. Gillespie DT (1977) Exact stochastic simulation of coupled chemical reactions. J Phys Chem 81: 2340–2361.
16. Nicolis G (1972) Fluctuations around nonequilibrium states in open nonlinear systems. J Stat Phys 6: 195–222.
17. Ueda HR, Hagiwara M, Kitano H (2001) Robust oscillations within the interlocked feedback model of drosophila circadian thythm. J theor Biol 210: 401–406.

18. Kusakina J, Dodd AN (2012) Phosphorylation in the plant circadian system. Trends in Plant Science : to be published.
19. Nagoshi E, Saini C, Bauer C, Laroche T, Naef F, et al. (2004) Circadian gene expression in individual fibroblasts: Cell-autonomous and self-sustained oscillatorspass time to daughter cells. Cell 119: 693–705.
20. Mihalcescu I, Hsing W, Leibler S (2004) Resilient circadian oscillator revealed in individual cyanobacteria. Nature 430: 81–85.
21. Amdaoud M, Vallade M, Weiss-Schaber C, Mihalcescu I (2007) Cyanobacterial clock, a stable phase oscillator with negligible intercellular coupling. PNAS 104: 7051–7056.
22. Liu C, Weaver DR, Strogatz SH, Reppert SM (1997) Cellular construction of a circadian clock: Period determination in the suprachiasmatic nuclei. Cell 91: 855–860.
23. Yamaguchi S, Isejima H, Matsuo T, Okura R, Yagita K, et al. (2003) Synchronization of cellular clocks in the suprachiasmatic nucleus. Science 302: 1408–1412.
24. Herzog ED, Aton SJ, Numano R, Sakaki Y, Tei H (2004) Temporal precision in the mammalian circadian system: A reliable clock from less reliable neurons. J Biol Rhythms 19: 35–46.
25. Carr AJF, Whitmore D (2005) Imaging of single light-responsive clock cells reveals uctuating free-running periods. Nature Cell Biology 7: 319–321.
26. Zeng H, Qian Z, Myers MP, Rosbash M (1996) A light-entrainment mechanism for the drosophila circadian clock. Nature 380: 129–135.
27. Leloup JC, Goldbeter A (1998) A model for circadian rhythms in drosophila incorporating the formation of a complex between the per and tim proteins. J Biol Rhythms 13: 70–87.
28. Glossop NRJ, Lyons LC, Hardin PE (1999) Interlocked feedback loops within the drosophila circadian oscillator. Science 286: 766–768.
29. Blau J (2001) The drosophila circadian clock: what we know and what we donft know. Semin Cell Dev Biol 12: 287–293.
30. Tomita J, Nakajima M, Kondo T, Iwasaki H (2005) No transcription-translation feedback in circadian rhythm of kaic phosphorylation. Science 307: 251–254.
31. Hirata H, Yoshiura S, Ohtsuka T, Bessho Y, Harada T, et al. (2002) Oscillatory expression of the bhlh factor hes1 regulated by a negative feedback loop. Science 298: 840–843.
32. Geva-Zatorsky N, Rosenfeld N, Itzkovitz S, Milo1 R, Sigal A, et al. (2006) Oscillations and variability in the p53 system. Molecular Systems Biology 2.
33. Elowitz MB, Leibler S (2000) A synthetic oscillatory network of transcriptional regulators. Nature 403: 335–338.
34. Atkinson MR, Savageau MA, Myers JT, Ninfa AJ (2003) Development of genetic circuitry exhibiting toggle switch or oscillatory behavior in escherichia coli. Cell 113: 597–607.
35. Tigges M, Marquez-Lago TT, Stelling J, Fussenegger M (2009) A tunable synthetic mammalian oscillator. Nature 457: 309–312.
